# *Lactobacillusplantarum* GKM3 and *Lactobacillus paracasei* GKS6 Supplementation Ameliorates Bone Loss in Ovariectomized Mice by Promoting Osteoblast Differentiation and Inhibiting Osteoclast Formation

**DOI:** 10.3390/nu12071914

**Published:** 2020-06-28

**Authors:** Li-Chan Yang, Shih-Wei Lin, I-Chen Li, Yen-Po Chen, Shih-Yang Tzu, Wei Chou, Chin-Chu Chen, Wen-Chuan Lin, Yen-Lien Chen, Wen-Hsin Lin

**Affiliations:** 1 Department of Pharmacy, China Medical University, Taichung 404, Taiwan; yang@mail.cmu.edu.tw (L.-C.Y.); wclin@mail.cmu.edu.tw (W.-C.L.); 2 Biotech Research Institute, Grape King Bio Ltd., Taoyuan City 325, Taiwan; wei.lin@grapeking.com.tw (S.-W.L.); ichen.li@grapeking.com.tw (I.-C.L.); yp.chen@grapeking.com.tw (Y.-P.C.); yt.shih@grapeking.com.tw (S.-Y.T.); wayne.chou@grapeking.com.tw (W.C.); gkbioeng@grapeking.com.tw (C.-C.C.); 3 Department of Food Science, Nutrition, and Nutraceutical Biotechnology, Shih Chien University, Taipei City 104, Taiwan; 4 Institute of Food Science and Technology, National Taiwan University, Taipei City 106, Taiwan; 5 Department of Bioscience Technology, Chung Yuan Christian University, Taoyuan City 320, Taiwan

**Keywords:** *Lactobacillus plantarum* GKM3, *Lactobacillus paracasei* GKS6, osteoporosis, osteoblast, osteoclast

## Abstract

Osteoporosis, an imbalance in the bone-forming process mediated by osteoblasts and the bone-resorbing function mediated by osteoclasts, is a bone degenerative disease prevalent among the aged population. Due to deleterious side effects of currently available medications, probiotics as a potential treatment of osteoporosis is an appealing approach. Hence, this study aims to evaluate the beneficial effects of two novel *Lactobacilli* strain probiotics on bone health in ovariectomized (OVX) induced osteoporotic mice model and its underlying mechanisms. Forty-five 9-week-old Institute of Cancer Research (ICR) mice underwent either a sham-operation (*n* = 9) or OVX (*n* = 36). Four days after the operation, OVX mice were further divided into four groups and received either saline alone, *Lactobacillus plantarum* GKM3, *Lactobacillus paracasei* GKS6 or alendronate per day for 28 days. After sacrifice by decapitation, right distal femur diaphysis was imaged via micro-computed tomography (MCT) and parameters including bone volume/tissue volume ratio (BV/TV), trabecular thickness (Tb.Th), trabecular number (Tb.N), trabecular separation (Tb.Sp), and bone mineral density (BMD) were measured. Moreover, GKM3 and GKS6 on RANKL-induced osteoclast formation and osteoblast differentiation using in vitro cultures were also investigated. The results showed that both probiotics strains inhibited osteoporosis in the OVX mice model, with *L. paracasei* GKS6 outperforming *L. plantarum* GKM3. Besides this, both GKS6 and GKM3 promoted osteoblast differentiation and inhibited RANKL-induced osteoclast differentiation via the Bone Morphogenetic Proteins (BMP) and RANKL pathways, respectively. These findings suggested that both strains of *Lactobacilli* may be pursued as potential candidates for the treatment and management of osteoporosis, particularly in postmenopausal osteoporosis.

## 1. Introduction

Osteoporosis is a widespread bone disease that is most prevalent among older people and postmenopausal women. Although the occurrence of the disease is not limited to this demographic, it has become increasingly problematic following the increase in life expectancy and the aging populations of present-day society, contributing to increased medical cost and public concern [1,2]. Characteristics of osteoporosis include a decrease in bone mass and deterioration of bone tissue microarchitecture, consequently leaving the patient susceptible to fracture due to bone fragility [2]. The crucial governing factor of bone integrity in humans is the homeostasis between bone-forming and bone-resorbing, facilitated by osteoblasts and osteoclasts, respectively. Once the balance is tipped in favor of increased osteoclastic resorption, as a result of menopausal estrogen deficiency in women, for example, the unbalanced rate of osteoblastic formation results in osteoporosis symptoms such as loss of bone mass and bone strength [3]. Therefore, therapies to promote osteoblast differentiation and inhibit osteoclast formation is an important therapeutic strategy.

In osteoblast differentiation, extracellular bone morphogenetic protein-2 (BMP-2) regulates the downstream osteogenic runt-related transcription factor2 (Runx2), which triggers the expression of osteoblastic markers such as alkaline phosphatase (ALP), osteopontin (OPN), and osteocalcin (OCN) [4]. In turn, osteoclast differentiation requires the binding of receptor activator of nuclear factor-κB (NF-κB) ligand (RANKL) via its receptor RANK located on the osteoclast membrane [5]. The interaction of RANKL to RANK then activates tumor necrosis factor receptor-associated factor 6 (TRAF6) which triggers key transcription factors for osteoclastogenesis, such as c-Fos and nuclear factor of activated T-cells cytoplasmic 1 (NFATc1) [6]. Of note, as a master regulator of osteoclastogenesis, NFATc1 can directly control osteoclast specific genes such as tartrate-resistant acid phosphatase (TRAP), cathepsin K (CTK), calcitonin receptor (CTR) and carbonic anhydrase II (CAII) [7].

Current pharmacological approaches to osteoporosis treatment include antiresorptive drugs which lower the rate of bone resorption such as bisphosphonates (alendronate [8,9], risedronate [10]) and estrogen agonists/antagonists (Raloxifene [11], Bazedoxifene [12]). However, various side effects after long-term usage of bisphosphonates are associated with jaw osteonecrosis and estrogen agonists can cause breast cancer and ovarian cancer have been reported [13,14]. Hence, new approaches with fewer side effects to promote bone health are needed.

The health benefits of probiotics, which are defined as live microbial dietary supplements that benefit the host animal by improving the microbial balance of gut flora, have been extensively researched recently. Interest in genus *Lactobacilli* in particular has grown exponentially in recent decades [15,16]. Studies have demonstrated putative beneficial effects of *Lactobacilli* probiotics in fields ranging from gastrointestinal to immunological disorders and obesity [17,18,19,20,21]. With regards to bone health, *L. casei* and *L. acidophilus,* as probiotic supplements, were fed to ovariectomized (OVX) mice, which are a well-established model used to simulate the estrogen drop-off condition in postmenopausal women [22,23,24]. After treatment, bone formation was shown to improve while bone resorption was reduced [25]. In another study, OVX mice were fed soy skim milk fermented with *L. paracasei* and *L. plantarum* as starters. Results suggested that the reduction in bone loss was due to the increase in aglycone isoflavones, soluble calcium, and vitamin D_3_ in the milk [26]. Compared to previous studies, this study focuses on two particular novel strains of *Lactobacilli, Lactobacillus plantarum* strain GKM3 and *Lactobacillus paracasei* strain GKS6, to evaluate their functional effects on osteoporosis in OVX mice model when the two are prepared without soy skim milk. To the best of our knowledge, this is the first time the effects of *Lactobacillus plantarum* strain GKM3 and *Lactobacillus paracasei* strain GKS6 on bone health and their possible mechanisms have been determined.

## 2. Materials and Methods

### 2.1. Preparation of Samples

*Lactobacillus plantarum* GKM3 strain from fresh vegetable and *Lactobacillus paracasei* GKS6 strain from infant feces were isolated by Grape King Bio Ltd., Taoyuan, Taiwan and are respectively preserved at the Bioresource Collection and Research Center (BCRC) of the Food Industry Research and Development Institute (FIRDI) with the preservation numbers of BCRC 910,787 and BCRC 910788. The cultivation of both strains of probiotic *Lactobacilli* was started in MRS broth (BD Difco, Sparks, MD, USA), then subcultured into 1.2 L of MRS broth inside a 2 L flask at 37 °C. The culture was then scaled up to 80% working volume of a 5-ton fermentor using synthetic medium (5% glucose, 2.0% yeast extract, 0.05% MgSO_4,_ 0.1% K_2_HPO_4_ and 0.1% Tween 80, pH 6.0) at 37 °C for 1 day. For in vitro cell culture, the final GKM3 and GKS6 liquid cultures were adjusted to pH 7 and then added onto MG-63 osteoblast-like cells or RAW 264.7 macrophages. For animal studies, the final liquid culture was freeze-dried into a powder, which was then prepared as a liquid feed by grinding and dissolving in 0.5% carboxymethylcellulose (CMC) to obtain suspensions at 20.5 mg/mL concentration. Before solvation, probiotic powder possessed cell counts of greater than, or equal to, 2 × 10^11^ colony-forming unit (CFU)/g. The positive control drug alendronate was dissolved in 0.5% CMC to obtain suspension at 0.25 mg/mL. All test samples were orally administered and performed according to a ratio of 0.1 mL liquid suspension per 10 g mouse body weight.

### 2.2. Animal Care and Handling

Eight-week old ICR female mice were purchased from BioLASCO, Taipei, Taiwan, and were housed for 7 days before the experiment. All mice were maintained in sanitary ventilated animal rooms (25 ± 2 °C) with a regular light cycle (12 h light: 12 h dark) and fed on standard lab diet ad libitum. Ovariectomy was performed at 9 weeks of age. Under anesthesia, double dorsolateral skin incisions were performed to remove the ovaries. In the sham-operated group, identical incisions were made but the ovaries were not removed. Upon sacrifice, the ovarian tissue was examined to confirm the success of ovariectomy. Experiment results were not used for mice where ovariectomy failed. All animal experiments were conducted in accordance with the current ethical regulations for animal care and use, and the protocol was approved by the Institute Animal Care and Use Committee (IACUC) of China Medical University (No. 2017-354).

Forty-five mice were randomly divided into one sham-control group and four ovariectomized (OXV) groups. In the four OVX groups, one group was treated with CMC as a control group, one was treated with the drug alendronate at 2.5 mg/kg as a positive control, and the remaining two groups were orally treated with GKM3 and GKS6 both a dose of 20.5 mg/kg. Administration of treatment solution was performed once per day, starting from 4 days post-operation, lasting for 28 consecutive days in total. Administration of alendronate was performed three times every week. The mice were sacrificed via decapitation 4 weeks after administration of test supplements, and the femur was collected for analysis.

### 2.3. Bone Tissue Analysis

Right distal femur diaphysis was imaged via micro-computed tomography (MCT) (SkyScan 1076, Kontizh, Belgium) at 18 μm resolution with the examiner blinded to the expected results. Upon examination and analysis via software of the images, bone morphometric parameters including ratio of bone volume to tissue volume (BV/TV), trabecular thickness (Tb.Th), trabecular number (Tb.N), and trabecular separation (Tb.Sp) were measured. The region 100 slices away from the distal femur growth plate was selected as the region of interest, not including cortical bone. Bone mineral density (BMD) was also measured in this selected region.

### 2.4. Cell Culture and Differentiation

MG-63 osteoblast-like cells and RAW 264.7 macrophages purchased from Bioresource Collection and Research Center (BCRC, Hsinchu, Taiwan) were cultured in Minimal Essential Medium (MEM) and Dulbecco’s modified Eagle's medium (DMEM) containing 10% fetal bovine serum (FBS) and 1% penicillin-streptomycin at 37 °C in a 5% CO_2_ incubator. The medium was changed every 2–3 days. Exposure concentrations for GKM3 and GKS6 were selected following cell viability tests performed. For osteoblast differentiation [27], MG-63 cells (2 × 10^5^ cells/well) were cultured in six wells for 24 h and changed to an MEM medium containing 1% FBS in the presence of 5% *v/v* GKM3 or GKS6 for the next 6 days. To induce osteoclast differentiation [28], a murine macrophage cell line, RAW 264.7 (2 × 10^5^ cells/well) were cultured in complete DMEM medium for 24 h and then supplemented with RANKL (50 ng/mL) in the presence of 5% *v/v* GKM3 or GKS6 for the next 6 days in a 6-well plate. A total of 2 ng/mL estradiol was used as a positive control. After culturing for 7 days, cells were harvested for subsequent analysis.

### 2.5. Total RNA Extraction and Quantitative PCR (Q-PCR) 

RNA is purified using the GeneJET RNA purification kit (Thermo Scientific, Waltham, MA, USA) and 1 µg of RNAs were reverse-transcribed with iScript™ cDNA Synthesis Kit (Bio-Rad, Hercules, CA, USA) following manufacturer’s instructions. The quantitative PCR cycling conditions were performed with initial denaturation of 95 °C for 5 min, followed by 40 cycles of denaturation (30 s) at 95 °C and amplification (30 s) at 60 °C in Bio-Rad CFX96 qPCR instrument (Bio-Rad, Hercules, CA, USA), using iTaq Universal SYBR Green Supermix (Bio-Rad, Hercules, CA, USA). Relative mRNA expression level was were normalized to GAPDH expression and the ∆∆Ct method was used for quantification. All reactions were run in triplicate and the target primer sequences are listed in Table 1.

### 2.6. Statistical Analysis

All data are presented as mean  ±  SD. Data analysis of this study was performed using one-way analysis of variance and Duncan’s multiple range test. Statistical results are labeled using lower-case alphabet letters where data labeled with the same letter denote no significant difference between groups. *p*-value <0.05 is considered statistically significant.

## 3. Results

### 3.1. Effects of Probiotics on Body Weight of OVX Mice

**Figure 1:**
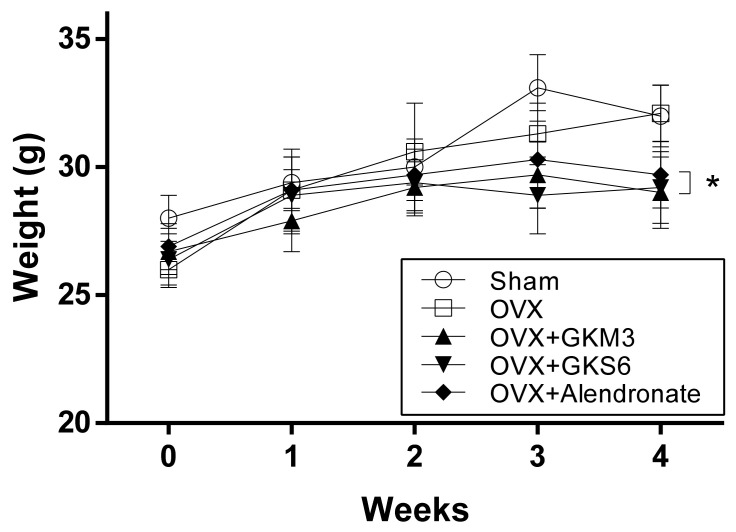
Body weight changes of mice in different groups. * *p* < 0.05 compared with ovariectomized (OVX) mice.

The body weight of OVX mice after the operation was found to be significantly lower than that of sham-control group at week 0 (*p* < 0.05; Figure 1). No significant differences were observed among the OVX mice at week 1 and week 2. While the weight of the untreated OVX group was able to return to the level of sham-controls after 4 weeks of treatment, GKM3, GKS6 or alendronate supplementation, however, attenuated the weight gain in OXV mice (*p* < 0.05; Figure 1). 

### 3.2. Effects of Probiotics on Bone Morphometric Parameters

**Figure 2:**
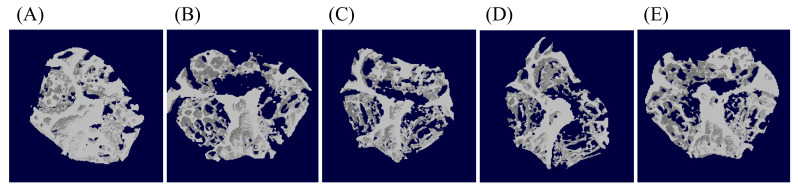
Representative Micro Computed Tomography images of Femur in (A) Sham group, (B) OVX untreated group, (C) OVX + GKM3 20.5 mg/kg group, (D) OVX + GKS6 20.5 mg/kg group and (E) OVX + alendronate 2.5 mg/kg group.

The representative MCT images of each group are shown in Figure 2. After 4 weeks of treatment, the BV/TV, Tb.N and Tb.Th values the untreated OVX group were found to be significantly lower than those of the sham-control group, whereas the value of Tb.Sp was higher (*p* < 0.05; Table 2). No significant differences in BV/TV and Tb.N values of the right femur were found among OVX groups. However, in groups supplemented with GKS6 and with alendronate, the Tb.Th and BMD values were significantly higher than those of the untreated OVX group (*p* < 0.05), while GKM3 supplementation displayed no significant difference compared with the untreated OVX group (*p* > 0.05). Nevertheless, all treatment groups had significantly less Tb.Sp at the right femur compared with that of the untreated OVX group (*p* < 0.05). 

### 3.3. Effect of Probiotics on Bone Metabolism-Related Gene Expressions

**Figure 3:**
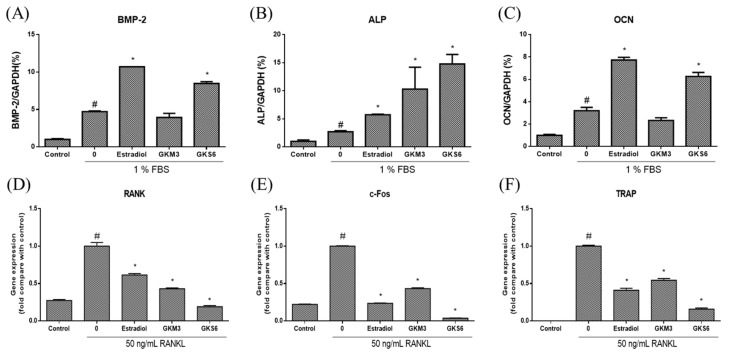
Effect of probiotics on osteoblast and osteoclast differentiation. Data are presented as the mean ± SD. For osteoblast differentiation, MG-63 cells were treated with estradiol, GKM3, and GKS6 in 1% FBS medium for 7 days. Gene expression of (A) bone morphogenetic protein-2 (BMP-2), (B) alkaline phosphatase (ALP), and (C) osteocalcin (OCN) on day 7 were determined by Q-PCR. For osteoclast differentiation, RAW264.7 were treated with estradiol, GKM3, and GKS6 in the presence of 50 ng/mL RANKL for 7 days. Gene expression of (D) receptor activator of nuclear factor-κB (RANK), (E) Cellular Oncogene Fos (c-Fos), and (F) tartrate-resistant acid phosphatase (TRAP) on day 7 were determined by Q-PCR. All data were normalized to GADPH. #*p* < 0.05 compared with control and *p < 0.05 compared with untreated.

Q-PCR was employed to measure mRNA levels of osteoblastic marker genes, including BMP-2, ALP, and OCN in 1% FBS-treated MG-63 cells. Estradiol was used as a positive control. Results showed that when compared with the untreated group (1% FBS induction), the mRNA levels of these genes in MG-63 cells were significantly increased after exposure to GKS6 (*p* < 0.05; Figure 3A–C). However, only ALP was significantly up-regulated in GKM3-treated group when compared with the corresponding untreated group (*p* < 0.05; Figure 3B).

On the other hand, RAW264.7 cells were cultured in presence of RANKL to examine the effect of GKM3 and GKS6 on osteoclastogenesis. As shown in Figure 3, the expression of RANK, c-fos, and TRAP was increased by RANKL treatment, but GKM3 and GKS6 treatment significantly inhibited the expression of these genes (*p* < 0.05; Figure 3D–E).

## 4. Discussion

Various studies on the supplementation of probiotics to reinforce bone health have recently been published in both healthy and pathological models, with *Lactobacilli* strains being the most commonly used [25,29,30]. The studies of Pan et al. showed beneficial effects both in OVX and aging models [26,31]. A key aspect of their study is the fermentation with soy skim milk instead of sole administration of probiotics. Thus, the effect of isoflavones from the soy skim milk cannot be ignored [26,31]. In this study, the liquid culture of two novel strains of *Lactobacillus*, *L. plantarum* GKM3 and *L. paracasei* GKS6 were instead collected and administered without fermentation with soy skim milk, such that their beneficial effects, when administered alone, may be evaluated. 

OVX mice were fed liquid resuspensions of freeze-dried whole liquid culture of two novel strains *L. plantarum* GKM3 and *L. paracasei* GKS6, to study the benefits of probiotics on bone physiology in an estrogen-deficient osteoporosis model. We first assess the effect of OVX-induced osteoporosis on weight changes in OVX mice (Figure 1). OVX in mice has been characterized to cause lowered metabolic rate and locomotor activity due to hormone imbalance, leading to weight gain. This aberrant weight gain can be prevented by administering estradiol [32]. In a study on the beneficial effects of the flavonoid glycoside naringin on OVX mice, Pang et al. attributed the lower body weight observed in groups treated with naringin to its effect of mimicking the activity of estrogen, as percent body weight gain in naringin-treated mice was statistically the same as estradiol-treated mice [33]. In our study, untreated OVX mice only gained marginally more weight than sham-control mice throughout the study period. However, a similar weight-gain-suppressing trend is seen wherein the mice treated with *L. paracasei* GKS6 and *L. plantarum* GKM3 both gained noticeably less weight than untreated OVX mice. This implies that probiotics treatment confers metabolic, or even hormonal homeostasis, restorative effects equivalent to administering estradiol. This is not a stretch, as Ostadmohammadi et al. have also shown that probiotics, in their case a mixture of four different strains co-supplemented with vitamin D, affected hormonal balance [34]. Blood tests revealed reduced serum testosterone levels as well as increased total antioxidant capacity in women with polycystic ovary syndrome (PCOS) after treatment with probiotics [34]. 

In a study similar to this paper, Ohlsson et al. also used *L. paracasei* and *L. plantarum* as probiotic feed for mice, wherein treatment started from 2 weeks prior to surgery and lasted until 4 weeks post-surgery in both OXV and sham-control groups. *L. paracasei* alone or mixed with *L. plantarum* was used [35]. The aim was to study the preventive effects of the probiotic by comparing bone characteristics between OVX and sham-control groups. However, while cortical bone characteristics such as bone mineral content (BMC) and cross-sectional bone area were protected, trabecular bone parameters were not [35]. In this study, BV/TV was significantly lower in OVX-untreated mice, indicating the loss of bone tissue relative to other body tissues. Furthermore, Tb.Th, Tb.N and BMD were computed to be lower while Tb.Sp was computed to be greater in OVX untreated mice, evidence of weakened bone microarchitecture. However, *L. paracasei* GKS6 treatment was able to maintain Tb.Th and BMD to a degree comparable to alendronate (Table 2). Although *L. plantarum* GKM3 treatment did not show significantly higher Tb.Th and BMD as compared to the untreated OVX group, all treated groups restored Tb.Sp to the level of the sham-control group. Comparing the two *Lactobacilli* strains used in this study, it is clear that *L. paracasei* GKS6 yielded better results across the table than *L. plantarum* GKM3. These findings suggest that bone volume and structural integrity were compromised after probiotics are supplemented, especially from *L. paracasei* GKS6. 

The related mechanism of the positive correlation between probiotics intake and osteoporosis amelioration is further investigated in vitro. BMP-2, a transcription factor, represents a major signaling pathway for regulating osteoblast differentiation and promoting bone formation [36]. Once activated, it translocates into the nucleus to enhance the activity of ALP, OCN as well as collagen synthesis [4]. In this study, we found that both GKM3 and GKS6 significantly increased the mRNA levels of BMP-2, ALP, and OCN, suggesting that both GKM3 and GKS6 may serve to promote osteoblastic differentiation in MG-63 cells. On the other hand, the differentiation and activation of osteoclasts are induced upon the binding of RANKL to RANK [37]. The interaction between RANK and RANKL results in the recruitment of c-Fos and NFATc1, regulating osteoclastogenesis-related genes such as TRAP. The results demonstrated that both GKM3 and GKS6 treatment significantly down-regulate the expression of RANK and c-Fos in the RANK signaling pathway and inhibit the expression of osteoclast-related genes, TRAP, suggesting that both GKM3 and GKS6 may serve to inhibit osteoclast differentiation in RAW264.7 cells. However, even though MG-63 and RAW264.7 cells are very useful for preliminary experiments, future studies should include histological analysis or the use of primary murine bone marrow cells, which could further increases translation and make results more physiologically relevant.

This study is the first to demonstrate that both GKM3 and GKS6 promote osteoblastic differentiation and inhibit osteoclastic differentiation through BMP and RANKL pathways, respectively. However, the limitation of this study is the lack of dose-dependent assay, which is important and must be explored in the future if a stronger correlation between probiotics and osteoporosis is to be asserted [38]. The identification of active compounds produced by GKM3 and GKS6 in ameliorating bone loss may also be conducted in future studies.

## 5. Conclusions

We have successfully shown that treatment of OVX-induced osteoporosis in mice with probiotic strains *L. plantarum* GKM3 and *L. paracasei* GKS6 maintains the integrity of bone microarchitecture, while GKS6 is slightly better than GKM3 in terms of efficacy measured across this study. Both GKM3 and GKS6 promote osteoblast differentiation and inhibit osteoclast formation via BMP and RANKL pathways, respectively. However, dose-dependent and active compound identification studies will potentially reveal stronger correlation and causation in the future.

## Figures and Tables

**Table 1:** Specific primers used for QPCR.

Gene	Forward	Reverse
BMP-2	5′−GGGTTGGAACTCCAGACTGT−3′	5′−GAAGAGTGAGTGGACCCCAG−3′
ALP	5′−CCACGTCTTCACATTTGGTG−3′	5′−AGACTGCGCCTAGTAGTTGT−3′
OCN	5′−TGAGAGCTCTCACACTCCTCGCCCTATTGG−3′	5′−GCTCCCAGCCATCGATACAGGTAGCGC−3′
RANK	5′−AAACCTTGGACCAACTGCAC−3′	5′−ACCATCTTCTCCTCCCHAGT−3′
c-Fos	5′−ATGGGCTCTCCTGTCAACAC−3′	5′−GGCTGCCAAAATAAACTCCA−3′
TRAP	5′−ACTTCCCCAGCCCTTACTACCG−3′	5′−TCAGCACATAGCCCACACCG−3′
GAPDH	5′−ACTTTGTCAAGCTCATTTCC−3′	5′−TGCAGCGAACTTTATTGATG−3′
BMP-2: bone morphogenetic protein-2; ALP: alkaline phosphatase; OCN: osteocalcin; RANK: receptor activator of nuclear factor-κB; TRAP: tartrate-resistant acid phosphatase		

**Table 2:** Effects of probiotics on bone morphometric parameters.

Treatments	Dose (mg/Kg)	BV/TV (%)	Tb.Th	Tb.N	Tb.Sp	BMD
			(μm)	(No./mm)	(μm)	(g/cm^3^)
Sham	-	40.9 ± 1.6 ^d^	112.6 ± 2.6 ^d^	3.58 ± 0.17 ^b^	282.2 ± 56.0 ^ab^	0.67 ± 0.05 ^d^
OVX	-	32.0 ± 2.1 ^a^	100.1 ± 6.6 ^a^	3.11 ± 0.16 ^a^	379.2 ± 51.8 ^c^	0.51 ± 0.03 ^a^
+GKM3	20.5	32.5 ± 2.3 ^ab^	104.6 ± 3.5 ^abc^	3.14 ± 0.13 ^a^	337.7 ± 44.7 ^ab^	0.55 ± 0.05 ^ab^
+GKS6	20.5	32.9 ± 2.7 ^abc^	106.9 ± 5.2 ^bcd^	3.11 ± 0.12 ^a^	288.4 ± 37.6 ^ab^	0.58 ± 0.02 ^bc^
+Alen	2.5	34.0 ± 2.1 ^abc^	106.9 ± 5.6 ^bcd^	3.20 ± 0.14 ^a^	255.1 ± 41.3 ^a^	0.59 ± 0.03 ^bc^

All data are expressed as mean ± SD (*n* = 9). Letters a, b, c, and d are used to express analysis results where data labeled with the same letter are not significantly different from each other (*p >* 0.05). Alen: alendronate; BV/TV: ratio of bone volume/tissue volume; Tb.Th: trabecular thickness; Tb.N: trabecular number; Tb.Sp: trabecular separation; and BMD: bone mineral density.
